# 2735. Treatment patterns and clinical outcomes of acyclovir resistant and refractory human herpes simplex virus infection after allogeneic hematopoietic cell transplantation

**DOI:** 10.1093/ofid/ofad500.2346

**Published:** 2023-11-27

**Authors:** Yeon Joo Lee, Yuxuan Li, Gyuri Han, Gunjan Shah, Roni Tamari, Miguel Perales, Genovefa Papanicolaou

**Affiliations:** Memorial Sloan Kettering Cancer Center, New York, New York; Memorial Sloan Kettering Cancer Center, New York, New York; Memorial Sloan Kettering Cancer Center, New York, New York; Memorial Sloan Kettering Cancer Center, New York, New York; Memorial Sloan Kettering Cancer Center, New York, New York; Memorial Sloan Kettering, New York, NY; Memorial Sloan Kettering Cancer Center, New York, New York

## Abstract

**Background:**

Treatment options for mucocutaneous herpes simplex virus infections (HSV) refractory or resistant (r/r) to acyclovir (ACV) are limited. High-dose ACV continuous infusion, foscarnet (FCN), cidofovir (CDV), and/or topical agents have been used. We report treatment patterns and outcomes of r/r HSV after allogeneic hematopoietic cell transplantation (HCT).

**Methods:**

Ault HCT recipients with r/r HSV at MSKCC from 1/1/2013 through 7/31/2022 were analyzed. HSV was diagnosed by dermal PCR. Antiviral susceptibility was performed at ARUP laboratories (Salt Lake City, UT) at clinicians’ discretion. The 50% inhibitory concentration for ACV was 2 ug/mL and for FCN 100 ug/mL. Standard care prophylaxis was ACV 400mg orally twice daily. Refractory HSV was defined as mucocutaneous HSV and failure to improve clinically after at least 7 days of standard treatment doses ACV, famciclovir, or valacyclovir and required ≥7 days of ACV ≥30mg/kg over 24 hours, FCN or CDV ≥1 dose. Resistant HSV was defined as refractory plus phenotypic resistance to ACV or FCN.

**Results:**

Fifteen patients met the criteria for r/r HSV (HSV1 86.7%; HSV2 13.3%) (Table 1). Virologic diagnosis for HSV occurred at a median of 74 days (Interquartile range [IQR], 34 - 236) post-HCT. Clinical definition for r/r HSV was a median of 86 days (IQR, 35 - 245.5) of post-HCT. Twelve had confirmed ACV resistance. All with r/r HSV required hospitalization for parenteral antivirals. Twelve (80%) had resolution of lesions after a median of 35 days (IQR, 30.25 – 69.25) of treatment and 3 (20%) had persistent lesions. Ten (67%) developed recurrence (median 2 episodes, range 1-7) after ≥2 weeks of oral maintenance therapy. Antiviral treatment is shown in Figure 1. Five (33.3%) experienced FCN-related side effects. One received Pritelivir (PTV) under an expanded access program due to persistent lesions despite being on FCN and topical CDV.

**Figure:**
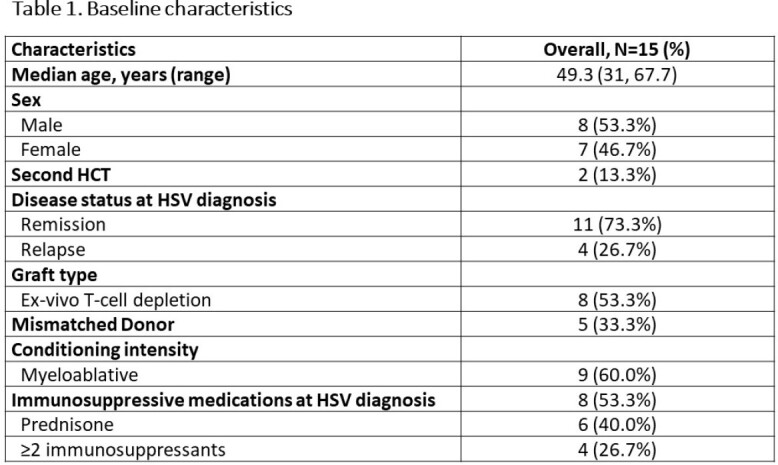

**Figure:**
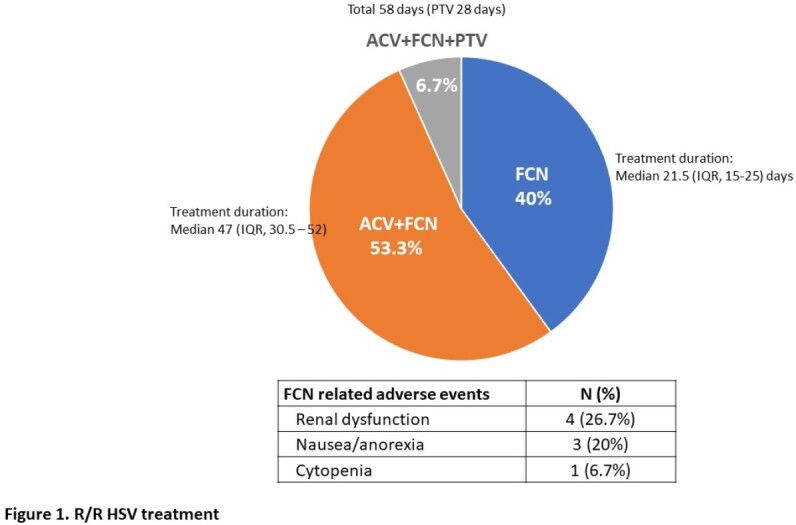

**Conclusion:**

One in 5 (20%) with r/r HSV failed to resolve with currently available therapies; 67% had a recurrence of r/r HSV after stopping treatment and 27% had nephrotoxicity due to FCN. One was treated successfully with PTV, an oral investigational antiviral without cross-resistance to ACV, FCN or CDV. Our data highlight the unmet need for effective, safe, and oral bioavailable antivirals for r/r HSV after HCT.

**Disclosures:**

**Yeon Joo Lee, MD, MPH**, AiCuris: institutional research support for clinical trials|Karius: institutional research support for clinical trials|Merck: Grant/Research Support|Scynexis: institutional research support for clinical trials **Miguel Perales, MD**, Adicet: Honoraria|Allogene: Honoraria|Allogene: institutional research support for clinical trials|Allovir: Honoraria|Bristol-Myers Squibb: Honoraria|Caribou Biosciences: Honoraria|Celgene: Honoraria|Cidara Therapeutics: Board Member|Equilium: Honoraria|Exevir: Honoraria|ImmPACT Bio: Honoraria|Incyte: Honoraria|Incyte: institutional research support for clinical trials|Karyopharm: Honoraria|Kite/Gilead: Honoraria|Kite/Gilead: institutional research support for clinical trials|Medigene: Board Member|Merck: Honoraria|Miltenyi Biotec: Honoraria|Miltenyi Biotec: institutional research support for clinical trials|MorphoSys: Honoraria|Nektar Therapeutics: Honoraria|Nektar Therapeutics: institutional research support for clinical trials|NexImmune: Board Member|NexImmune: Ownership Interest|Novartis: Honoraria|Novartis: institutional research support for clinical trials|Omeros: Honoraria|Omeros: Ownership Interest|OrcaBio: Honoraria|OrcaBio: Ownership Interest|Sellas Life Sciences: Board Member|Syncopation: Honoraria|VectivBio AG: Honoraria|Vor Biopharma: Honoraria **Genovefa Papanicolaou, MD**, Allovir: Advisor/Consultant|Amplyx: Advisor/Consultant|Astellas: Advisor/Consultant|Cidara: Advisor/Consultant|CSL Behring: Advisor/Consultant|DSMC: Advisor/Consultant|Merck: Advisor/Consultant|Merck: Grant/Research Support|Merck: institutional research support for clinical trials|MSD: Advisor/Consultant|Octapharma: Advisor/Consultant|Partners Rx: Advisor/Consultant|Shire/Takeda: institutional research support for clinical trials|Symbio: Advisor/Consultant|Symbio: Advisor/Consultant|Takeda: Advisor/Consultant|Vera Pharma: Advisor/Consultant

